# (*Z*,*Z*)-1,4-Diiodo-1,4-bis­(trimethyl­silyl)buta-1,3-diene

**DOI:** 10.1107/S160053680803482X

**Published:** 2008-10-31

**Authors:** Jan W. Bats, Birgit Urschel, Thomas Müller

**Affiliations:** a Institut für Organische Chemie, Universität Frankfurt, Max-von-Laue-Strasse 7, D-60438 Frankfurt am Main, Germany; b Institut für Anorganische Chemie, Universität Frankfurt, Max-von-Laue-Strasse 7, D-60438 Frankfurt am Main, Germany; c Institut für Reine und Angewandte Chemie, Universität Oldenburg, Carl-von-Ossietzky-Strasse 9-11, D-26129 Oldenburg, Germany

## Abstract

The asymmetric unit of the title compound, C_10_H_20_I_2_Si_2_, contains two half-mol­ecules.  Both complete molecules are generated by crystallographic inversion centers located at the mid-points of the central C—C single bonds; the butadiene groups are planar, with a *trans* conformation about the central C—C bond. The mol­ecules show short intra­molecular H⋯I contacts of 2.89 and 2.92 Å. The crystal packing shows no short inter­molecular contacts.

## Related literature

For the synthesis of the title compound, see: Yamaguchi *et al.* (1998 ▶). For related structures, see: Saito *et al.* (2007 ▶); Yamamoto *et al.* (2002 ▶). For van der Waals radii, see: Bondi (1964 ▶).
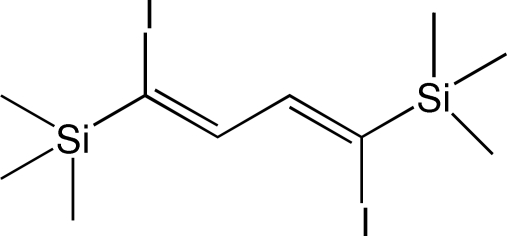


## Experimental

### 

#### Crystal data

C_10_H_20_I_2_Si_2_
*M* *_r_* = 450.24
Triclinic, 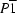

*a* = 6.3553 (17) Å
*b* = 11.502 (2) Å
*c* = 11.698 (2) Å
α = 103.027 (13)°
β = 90.555 (17)°
γ = 90.99 (2)°
*V* = 832.9 (3) Å^3^
*Z* = 2
Mo *K*α radiation
μ = 3.89 mm^−1^
*T* = 155 (2) K
0.46 × 0.36 × 0.28 mm

#### Data collection

Siemens SMART 1K CCD diffractometer
Absorption correction: multi-scan (*SADABS*; Sheldrick, 2000 ▶) *T*_min_ = 0.275, *T*_max_ = 0.336
15331 measured reflections
5837 independent reflections
5272 reflections with *I* > 2σ(*I*)
*R*_int_ = 0.021

#### Refinement

*R*[*F* ^2^ > 2σ(*F* ^2^)] = 0.021
*wR*(*F* ^2^) = 0.059
*S* = 1.03
5837 reflections
134 parameters
H-atom parameters constrained
Δρ_max_ = 1.12 e Å^−3^
Δρ_min_ = −0.94 e Å^−3^

### 

Data collection: *SMART* (Siemens, 1995 ▶); cell refinement: *SMART*; data reduction: *SAINT* (Siemens, 1995 ▶); program(s) used to solve structure: *SHELXS97* (Sheldrick, 2008 ▶); program(s) used to refine structure: *SHELXL97* (Sheldrick, 2008 ▶); molecular graphics: *SHELXTL* (Sheldrick, 2008 ▶); software used to prepare material for publication: *SHELXL97*.

## Supplementary Material

Crystal structure: contains datablocks global, I. DOI: 10.1107/S160053680803482X/su2074sup1.cif

Structure factors: contains datablocks I. DOI: 10.1107/S160053680803482X/su2074Isup2.hkl

Additional supplementary materials:  crystallographic information; 3D view; checkCIF report

## Figures and Tables

**Table 1:** Hydrogen-bond geometry (Å, °)

*D*—H⋯*A*	*D*—H	H⋯*A*	*D*⋯*A*	*D*—H⋯*A*
C1—H1⋯I1^i^	0.95	2.92	3.394 (2)	112
C6—H6⋯I2^ii^	0.95	2.89	3.378 (2)	113

Symmetry codes: (i) 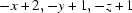; (ii) 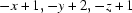.

**Fig. 1.**
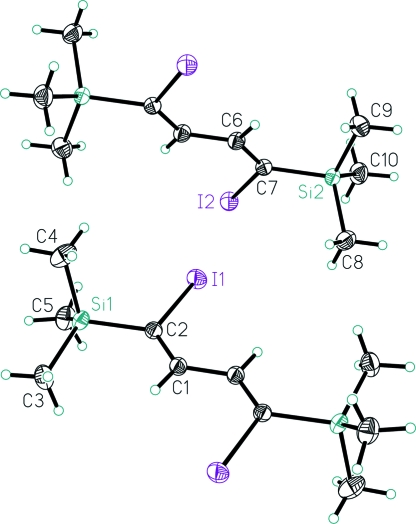
A view of the two independent molecules of the title compound, with displacement ellipsoids drawn at the 50% probability level (Unlabeled atoms are related to labeled atoms by inversion centers at the midpoints of the molecules).

**Fig. 2.**
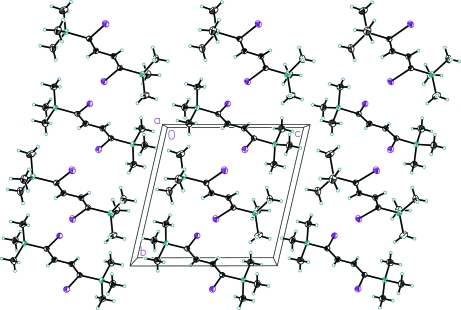
The crystal packing of the title compound, viewed down the a axis (the displacement ellipsoids are drawn at the 50% probability level).

## supplementary crystallographic information

### Comment

The title compound crystallized with two independent centrosymmetric molecules in the unit cell (Fig.1). Each molecule has a crystallographic inversion center at the midpoint of the central C—C single bond. The geometrical parameters of both molecules are similar. The butadiene groups are planar with a *trans*-conformation about the central C—C bond. The trimethylsilyl groups adopt orientations with a methyl group *syn*-periplanar with the nearest C=C double bond: torsion angles C3—Si1—C2—C1 = -12.1 (2)° and C9—Si2—C7—C6 = -21.2 (2)°. The molecules show intramolecular H···I contacts of 2.89 Å and 2.92 Å (Table 1), which are shorter than the van der Waals contact distance of 3.18 Å (Bondi, 1964).

The crystal packing of the title compound (Fig. 2) shows no short intermolecular contacts. The shortest intermolecular I···I distances of 3.876 (1) Å [I1···I2^i^; symmetry operation i) = 1+x, y, z] and 3.973 (1) Å [I1···I2] are comparable to the van der Waals contact distance of 3.96 Å.

### Experimental

The title compound was prepared as described by Yamaguchi *et al.* (1998), and recrystallized from n-hexane at 153 K.

### Refinement

H atoms were geometrically positioned and treated as riding atoms: C_planar_—H = 0.95 Å, C_methyl_—H=0.98 Å, with *U*_iso_(H)= 1.2*U*_eq_(C_butene_) and = 1.5*U*_eq_(C_methyl_).

### Crystal data

**Table:**

C_10_H_20_I_2_Si_2_	*Z* = 2
*M**_r_* = 450.24	*F*(000) = 428
Triclinic, *P*1	*D*_x_ = 1.795 Mg m^−^^3^
Hall symbol: -P 1	Mo *K*α radiation, λ = 0.71073 Å
*a* = 6.3553 (17) Å	Cell parameters from 124 reflections
*b* = 11.502 (2) Å	θ = 3–23°
*c* = 11.698 (2) Å	µ = 3.89 mm^−^^1^
α = 103.027 (13)°	*T* = 155 K
β = 90.555 (17)°	Block, colorless
γ = 90.99 (2)°	0.46 × 0.36 × 0.28 mm
*V* = 832.9 (3) Å^3^	

### Data collection

**Table:**

Siemens SMART 1K CCD diffractometer	5837 independent reflections
Radiation source: normal-focus sealed tube	5272 reflections with *I* > 2σ(*I*)
graphite	*R*_int_ = 0.021
ω scans	θ_max_ = 32.5°, θ_min_ = 1.8°
Absorption correction: multi-scan (SADABS; Sheldrick, 2000)	*h* = −9→9
*T*_min_ = 0.275, *T*_max_ = 0.336	*k* = −17→17
15331 measured reflections	*l* = −17→17

### Refinement

**Table:**

Refinement on *F*^2^	Secondary atom site location: difference Fourier map
Least-squares matrix: full	Hydrogen site location: inferred from neighbouring sites
*R*[*F*^2^ > 2σ(*F*^2^)] = 0.021	H-atom parameters constrained
*wR*(*F*^2^) = 0.059	*w* = 1/[σ^2^(*F*_o_^2^) + (0.02*P*)^2^] where *P* = (*F*_o_^2^ + 2*F*_c_^2^)/3
*S* = 1.03	(Δ/σ)_max_ = 0.002
5837 reflections	Δρ_max_ = 1.12 e Å^−^^3^
134 parameters	Δρ_min_ = −0.94 e Å^−^^3^
0 restraints	Extinction correction: SHELXL97 (Sheldrick, 2008), Fc^*^=kFc[1+0.001xFc^2^λ^3^/sin(2θ)]^-1/4^
Primary atom site location: structure-invariant direct methods	Extinction coefficient: 0.0130 (5)

### Special details

**Table:**

Geometry. All e.s.d.'s (except the e.s.d. in the dihedral angle between two l.s. planes) are estimated using the full covariance matrix. The cell e.s.d.'s are taken into account individually in the estimation of e.s.d.'s in distances, angles and torsion angles; correlations between e.s.d.'s in cell parameters are only used when they are defined by crystal symmetry. An approximate (isotropic) treatment of cell e.s.d.'s is used for estimating e.s.d.'s involving l.s. planes.
Refinement. Refinement of *F*^2^ against ALL reflections. The weighted *R*-factor *wR* and goodness of fit *S* are based on *F*^2^, conventional *R*-factors *R* are based on *F*, with *F* set to zero for negative *F*^2^. The threshold expression of *F*^2^ > σ(*F*^2^) is used only for calculating *R*-factors(gt) *etc*. and is not relevant to the choice of reflections for refinement. *R*-factors based on *F*^2^ are statistically about twice as large as those based on *F*, and *R*- factors based on ALL data will be even larger.

### Fractional atomic coordinates and isotropic or equivalent isotropic displacement parameters (Å^2^)

**Table:**

	*x*	*y*	*z*	*U*_iso_*/*U*_eq_	
I1	0.631493 (18)	0.679368 (10)	0.508829 (10)	0.02900 (4)	
I2	0.124665 (17)	0.825214 (9)	0.402449 (9)	0.02623 (4)	
Si1	0.76330 (7)	0.63323 (4)	0.77198 (4)	0.02223 (9)	
Si2	0.47343 (7)	0.86551 (4)	0.19545 (4)	0.02225 (9)	
C1	0.9715 (3)	0.51656 (14)	0.56130 (13)	0.0211 (3)	
H1	1.0502	0.4804	0.6129	0.025*	
C2	0.8224 (2)	0.59143 (13)	0.61143 (13)	0.0198 (3)	
C3	0.9030 (3)	0.53065 (18)	0.84764 (16)	0.0321 (4)	
H3A	0.8575	0.5446	0.9293	0.048*	
H3B	1.0552	0.5455	0.8458	0.048*	
H3C	0.8702	0.4478	0.8077	0.048*	
C4	0.8577 (4)	0.79022 (18)	0.8278 (2)	0.0405 (5)	
H4A	0.8270	0.8157	0.9116	0.061*	
H4B	0.7858	0.8422	0.7848	0.061*	
H4C	1.0098	0.7953	0.8165	0.061*	
C5	0.4740 (3)	0.62065 (18)	0.79288 (17)	0.0322 (4)	
H5A	0.4456	0.6250	0.8759	0.048*	
H5B	0.4206	0.5442	0.7457	0.048*	
H5C	0.4037	0.6862	0.7680	0.048*	
C6	0.5181 (3)	0.98312 (14)	0.43727 (13)	0.0221 (3)	
H6	0.6391	1.0180	0.4103	0.027*	
C7	0.4013 (3)	0.90828 (14)	0.35421 (13)	0.0210 (3)	
C8	0.6036 (3)	0.71790 (16)	0.16638 (17)	0.0332 (4)	
H8A	0.7252	0.7220	0.2194	0.050*	
H8B	0.6506	0.6974	0.0849	0.050*	
H8C	0.5034	0.6567	0.1796	0.050*	
C9	0.6592 (3)	0.98330 (18)	0.16797 (18)	0.0361 (4)	
H9A	0.7846	0.9870	0.2182	0.054*	
H9B	0.5901	1.0607	0.1859	0.054*	
H9C	0.7005	0.9641	0.0854	0.054*	
C10	0.2345 (3)	0.85504 (18)	0.09994 (16)	0.0342 (4)	
H10A	0.2759	0.8370	0.0174	0.051*	
H10B	0.1613	0.9312	0.1182	0.051*	
H10C	0.1404	0.7914	0.1139	0.051*	

### Atomic displacement parameters (Å^2^)

**Table:**

	*U*^11^	*U*^22^	*U*^33^	*U*^12^	*U*^13^	*U*^23^
I1	0.02999 (7)	0.03118 (7)	0.02837 (7)	0.01194 (4)	0.00029 (5)	0.01117 (5)
I2	0.02378 (7)	0.02802 (7)	0.02598 (6)	−0.00570 (4)	−0.00083 (4)	0.00471 (4)
Si1	0.0211 (2)	0.0241 (2)	0.01973 (19)	0.00282 (16)	0.00184 (15)	0.00115 (16)
Si2	0.0263 (2)	0.0217 (2)	0.01793 (19)	0.00165 (16)	0.00037 (16)	0.00277 (15)
C1	0.0221 (7)	0.0224 (7)	0.0191 (6)	0.0036 (5)	0.0002 (5)	0.0049 (5)
C2	0.0193 (7)	0.0203 (7)	0.0200 (6)	0.0019 (5)	−0.0005 (5)	0.0046 (5)
C3	0.0302 (9)	0.0438 (10)	0.0248 (8)	0.0096 (7)	0.0031 (7)	0.0123 (7)
C4	0.0426 (12)	0.0312 (9)	0.0408 (11)	−0.0023 (8)	−0.0044 (9)	−0.0063 (8)
C5	0.0224 (9)	0.0423 (10)	0.0307 (9)	0.0045 (7)	0.0053 (7)	0.0050 (7)
C6	0.0213 (7)	0.0223 (7)	0.0217 (7)	−0.0019 (5)	0.0011 (5)	0.0030 (5)
C7	0.0219 (7)	0.0203 (7)	0.0207 (7)	0.0013 (5)	0.0008 (5)	0.0045 (5)
C8	0.0414 (11)	0.0279 (9)	0.0284 (8)	0.0081 (7)	−0.0002 (7)	0.0021 (7)
C9	0.0410 (11)	0.0339 (9)	0.0349 (9)	−0.0020 (8)	0.0109 (8)	0.0108 (8)
C10	0.0387 (11)	0.0388 (10)	0.0245 (8)	0.0034 (8)	−0.0048 (7)	0.0060 (7)

### Geometric parameters (Å, °)

**Table:**

I1—C2	2.1207 (16)	C4—H4B	0.9800
I2—C7	2.1276 (17)	C4—H4C	0.9800
Si1—C3	1.8591 (19)	C5—H5A	0.9800
Si1—C4	1.863 (2)	C5—H5B	0.9800
Si1—C5	1.8647 (19)	C5—H5C	0.9800
Si1—C2	1.8742 (16)	C6—C7	1.350 (2)
Si2—C10	1.863 (2)	C6—C6^ii^	1.453 (3)
Si2—C8	1.8644 (19)	C6—H6	0.9500
Si2—C9	1.866 (2)	C8—H8A	0.9800
Si2—C7	1.8744 (16)	C8—H8B	0.9800
C1—C2	1.339 (2)	C8—H8C	0.9800
C1—C1^i^	1.450 (3)	C9—H9A	0.9800
C1—H1	0.9500	C9—H9B	0.9800
C3—H3A	0.9800	C9—H9C	0.9800
C3—H3B	0.9800	C10—H10A	0.9800
C3—H3C	0.9800	C10—H10B	0.9800
C4—H4A	0.9800	C10—H10C	0.9800
			
C3—Si1—C4	110.85 (10)	Si1—C5—H5A	109.5
C3—Si1—C5	109.79 (9)	Si1—C5—H5B	109.5
C4—Si1—C5	110.45 (10)	H5A—C5—H5B	109.5
C3—Si1—C2	109.04 (8)	Si1—C5—H5C	109.5
C4—Si1—C2	107.21 (9)	H5A—C5—H5C	109.5
C5—Si1—C2	109.45 (8)	H5B—C5—H5C	109.5
C10—Si2—C8	109.30 (9)	C7—C6—C6^ii^	128.06 (19)
C10—Si2—C9	110.53 (10)	C7—C6—H6	116.0
C8—Si2—C9	110.39 (10)	C6^ii^—C6—H6	116.0
C10—Si2—C7	110.61 (9)	C6—C7—Si2	123.88 (12)
C8—Si2—C7	108.94 (8)	C6—C7—I2	119.83 (12)
C9—Si2—C7	107.04 (8)	Si2—C7—I2	116.23 (8)
C2—C1—C1^i^	128.56 (19)	Si2—C8—H8A	109.5
C2—C1—H1	115.7	Si2—C8—H8B	109.5
C1^i^—C1—H1	115.7	H8A—C8—H8B	109.5
C1—C2—Si1	126.01 (12)	Si2—C8—H8C	109.5
C1—C2—I1	120.61 (12)	H8A—C8—H8C	109.5
Si1—C2—I1	113.35 (8)	H8B—C8—H8C	109.5
Si1—C3—H3A	109.5	Si2—C9—H9A	109.5
Si1—C3—H3B	109.5	Si2—C9—H9B	109.5
H3A—C3—H3B	109.5	H9A—C9—H9B	109.5
Si1—C3—H3C	109.5	Si2—C9—H9C	109.5
H3A—C3—H3C	109.5	H9A—C9—H9C	109.5
H3B—C3—H3C	109.5	H9B—C9—H9C	109.5
Si1—C4—H4A	109.5	Si2—C10—H10A	109.5
Si1—C4—H4B	109.5	Si2—C10—H10B	109.5
H4A—C4—H4B	109.5	H10A—C10—H10B	109.5
Si1—C4—H4C	109.5	Si2—C10—H10C	109.5
H4A—C4—H4C	109.5	H10A—C10—H10C	109.5
H4B—C4—H4C	109.5	H10B—C10—H10C	109.5
			
C1^i^—C1—C2—Si1	−178.07 (17)	C6^ii^—C6—C7—Si2	−176.55 (18)
C1^i^—C1—C2—I1	0.2 (3)	C6^ii^—C6—C7—I2	0.6 (3)
C3—Si1—C2—C1	−12.13 (17)	C10—Si2—C7—C6	−141.69 (15)
C4—Si1—C2—C1	107.93 (16)	C8—Si2—C7—C6	98.15 (16)
C5—Si1—C2—C1	−132.25 (15)	C9—Si2—C7—C6	−21.21 (17)
C3—Si1—C2—I1	169.49 (8)	C10—Si2—C7—I2	41.11 (11)
C4—Si1—C2—I1	−70.44 (11)	C8—Si2—C7—I2	−79.04 (11)
C5—Si1—C2—I1	49.37 (11)	C9—Si2—C7—I2	161.59 (9)

Symmetry codes: (i) −*x*+2, −*y*+1, −*z*+1; (ii) −*x*+1, −*y*+2, −*z*+1.

### Hydrogen-bond geometry (Å, °)

**Table:**

*D*—H···*A*	*D*—H	H···*A*	*D*···*A*	*D*—H···*A*
C1—H1···I1^i^	0.95	2.92	3.394 (2)	112
C6—H6···I2^ii^	0.95	2.89	3.378 (2)	113

Symmetry codes: (i) −*x*+2, −*y*+1, −*z*+1; (ii) −*x*+1, −*y*+2, −*z*+1.
